# Effects of including sprints during prolonged cycling on hormonal and muscular responses and recovery in elite cyclists

**DOI:** 10.1111/sms.13865

**Published:** 2020-11-07

**Authors:** Nicki Winfield Almquist, Stian Ellefsen, Øyvind Sandbakk, Bent R. Rønnestad

**Affiliations:** ^1^ Institute of Public Health and Sport Sciences Inland Norway University of Applied Sciences Lillehammer Norway; ^2^ Center for Elite Sports Research Department of Neuromedicine and Movement Science Norwegian University of Science and Technology Trondheim Norway

**Keywords:** 30‐sec sprints, aerobic and anaerobic fitness, blood hormones, Elite athletes, mRNA, muscular responses, prolonged low‐intensity cycling

## Abstract

This study investigated the acute effects of including 30‐second sprints during prolonged low‐intensity cycling on muscular and hormonal responses and recovery in elite cyclists. Twelve male cyclists (VO_2max_, 73.4 ± 4.0 mL/kg/min) completed a randomized crossover protocol, wherein 4 hours of cycling at 50% of VO_2max_ were performed with and without inclusion of three sets of 3 × 30 seconds maximal sprints (*E&S* vs *E,* work‐matched). Muscle biopsies (m. vastus lateralis) and blood were sampled at Pre, immediately after (Post) and 3 hours after (3 h) finalizing sessions. *E&S* led to greater increases in mRNA levels compared with *E* for markers of fat metabolism (PDK4, Δ‐Log2 fold change between *E&S* and *E* ± 95%CI Post; 2.1 ± 0.9, Δ3h; 1.3 ± 0.7) and angiogenesis (VEGFA, Δ3h; 0.3 ± 0.3), and greater changes in markers of muscle protein turnover (myostatin, ΔPost; −1.4 ± 1.2, Δ3h; −1.3 ± 1.3; MuRF1, ΔPost; 1.5 ± 1.2, all *P* < .05). *E&S* showed decreased mRNA levels for markers of ion transport at 3h (Na^+^‐K^+^ α1; −0.6 ± 0.6, CLC1; −1.0 ± 0.8 and NHE1; −0.3 ± 0.2, all *P* < .05) and blunted responses for a marker of mitochondrial biogenesis (PGC‐1α, Post; −0.3 ± 0.3, 3h; −0.4 ± 0.3, *P* < .05) compared with *E* *E&S* and *E* showed similar endocrine responses, with exceptions of GH and SHBG, where *E&S* displayed lower responses at Post (GH; −4.1 ± 3.2 μg/L, SHBG; −2.2 ± 1.9 nmol/L, *P* < .05). Both *E&S* and *E* demonstrated complete recovery in isokinetic knee extension torque 24 hours after exercise. In conclusion, we demonstrate *E&S* to be an effective exercise protocol for elite cyclists, which potentially leads to beneficial adaptations in skeletal muscle without impairing muscle recovery 24 hours after exercise.

## 1 INTRODUCTION

Elite cycling involves prolonged and strenuous competitions with distances up to 250‐300 km.^1^, ^2^ During such competitions, intensities vary from aerobic low‐intensity to all‐out sprinting, and preparations thus need to include development of both aerobic and anaerobic fitness.^3^ Accordingly, elite cyclists typically cover annual distances of ~ 26‐32 000 km and ~ 850 hours of training, wherein ~ 70%‐80% is low‐intensity training (LIT),^1^, ^4^ with singular sessions lasting up to 7 hours, interspersed by high‐intensity training.^2^ Given the extent of these training loads, there is no simple measure for further improving performance by altering training protocols. Simply increasing the duration of LIT does not seem to be sufficient.^5^ Instead, it seems necessary to increase the training volume at higher exercise intensities.^6^ This is achievable by implementation of sprint training, which improves both aerobic and anaerobic performances.^6^ However, for elite cyclists, it is not time‐efficient to dedicate singular sessions to sprint training, which also might increase the need for more restitution. A possible solution would be to include small volumes of sprint intervals during habitual LIT exercises.^7^, ^8^, ^9^, ^10^ This may improve performance in athletes that are already close to their genetic maximum.^11^ Previous studies have exclusively studied this by adding sprints during relatively short LIT sessions (1‐1.5 hours).^8^, ^9^, ^10^ For such training to be advocated for elite cyclists, it is important to assess its effects and feasibility during LIT sessions of regular duration (>3‐4 hours).^2^, ^7^ It seems expedient to start such an exploration by investigating its acute effects on muscular and hormonal adaptations, as well as the associated need for post‐exercise restitution.

In skeletal muscle, inclusion of 30‐second sprints during short‐lasting LIT session leads to acute increases in markers of mitochondrial function and biogenesis in muscle of trained subjects compared with LIT‐only.^8^, ^10^ This fits the notion that markers of mitochondrial biogenesis, such as peroxisome proliferator‐activated receptor gamma coactivator‐1α (PGC‐1α), respond to acute exercises in an intensity‐dependent fashion,^12^ making it a potential determinant of these beneficial effects. Furthermore, sprint training seems to increase abundances of ion transport‐proteins in muscle, thus augmenting ion transport capacity, which has been suggested to postpone fatigue development and improve high‐intensity endurance performance.^13^ To date, the effects of including sprints during prolonged LIT sessions on markers of ion transport in muscle remain unknown. Finally, sprint exercise may also affect protein turnover in skeletal muscle, as orchestrated through acute regulation of a range of muscular signaling factors such as PGC‐1α4, insulin‐like growth factor 1 (IGF1), and myostatin.^14^ Together, this may facilitate the development of oxidative muscle fibers and may be beneficial for the ability to develop maximal aerobic power and fractional utilization of VO_2max_.

Evidently, muscular responses to exercise largely reside on signaling events arising from increased muscular activation. However, such responses are also affected by systemic factors, including exercise‐associated changes in hormone levels in blood.^15^, ^16^ This perspective remains scarcely investigated after combined sprint and LIT exercise,^8^, ^9^, ^10^ though repeated maximal sprints alone lead to acute increases in testosterone, growth hormone (GH), and cortisol.^15^, ^17^ Testosterone has been suggested to increase muscle protein turnover, perhaps acting in concert with GH and insulin‐like growth factor 1 (IGF1).^18^ In line with this, testosterone may also be positive for recovery in endurance athletes^19^ and may act to increase plasma volume and erythropoiesis,^20^ subsequently leading to increased VO_2max_.^20^ Such growth and repair responses may also involve cortisol, which may be necessary for recycling proteins, resulting in a pool of free amino acids that may facilitate muscle protein synthesis during the restitution phase.^21^ Overall, inclusion of sprints during prolonged, habitual LIT sessions of elite cyclists (~4 hours) may provide an effective stimulus for inducing muscular and systemic plasticity in elite cyclists, a perspective that remains unstudied.

The aim of the present study was to investigate the acute effects of including repeated 30‐second sprints during a 4‐hour LIT session on muscular and hormonal responses and the subsequent recovery (peak knee extension torque) in elite cyclists. We hypothesized that adding sprints to LIT (*E&S*) would lead to increased mRNA responses for genes involved in mitochondrial function and biogenesis, angiogenesis, ion transport, and protein turnover in m. vastus lateralis, as well as increased hormonal responses, compared to LIT alone (*E*).

## 2 METHODS

### 2.1 Participants

Twelve male cyclists (26.2 ± 6.3 years of age) volunteered for the study. Average endurance training recorded the last 30 days preceding study inclusion amounted to 13 ± 8 hour/week of which 0.6 ± 0.4 hour/week was high‐intensity training. The participants did not perform sprint training on a regular basis. All participants were defined as elite cyclists, with nine participants being performance level 5 athletes and the remaining three participants being level 4 athletes, as classified using data on VO_2max_, maximal aerobic power produced during the last minute of an incremental test to exhaustion (*W*_max_) and training volume^22^ (Table 1). Prior to inclusion, participants were informed of the possible risks and discomforts associated with the study and provided written, informed consents to participate. The study was approved by the Norwegian Center for Research Data (NSD) and the local ethical committee at Inland Norway University of Applied Sciences and was performed according to the Declaration of Helsinki (except pre‐registration in public databases).

**TABLE 1:** Subject characteristics and physiological parameters at baseline

Body mass (kg)	76 ± 3
Height (cm)	183 ± 5
Power output at 4 mmol/L [Bla^‐^] (W/kg)	4.3 ± 0.6
VO_2max_ (mL/kg/min)	73.4 ± 4.0
W_max_ (W/kg)	6.3 ± 0.3
Wingate mean power output (W)	851 ± 64

Note

Maximal oxygen consumption (VO_2max_), maximal power produced the last minute during incremental test to exhaustion (*W*_max_), mean power output on a 30‐s all‐out sprint (Wingate). Values are mean ± standard deviation (SD), n = 12.

### 2.2 Experimental design and procedures

**FIGURE 1:**
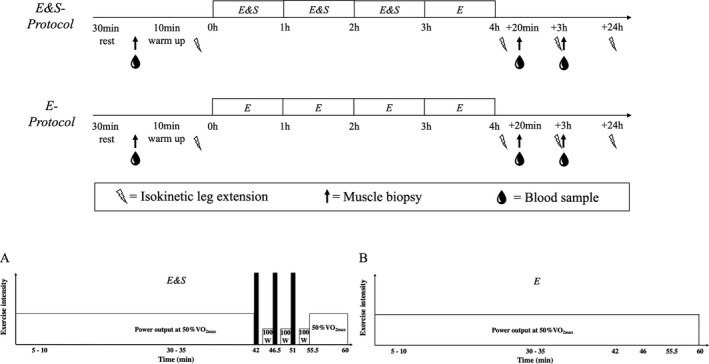
Overview of experimental protocols (two top panels) endurance exercise including sprints (E&S) and work‐matched endurance exercise (E) and detailed description of every hour in E&S (panel A) and E protocols (panel B). Lightnings symbolize isokinetic knee extension, black arrows symbolize muscle biopsy, and blood drops indicate blood sample

Data on acute physiological responses to the exercise protocol are published elsewhere,^7^ but for clarification, the design of this randomized, work‐matched, crossover study is briefly outlined here (Figure 1). Participants visited the laboratory on four occasions to perform (1) screening, (2) familiarization, and (3 + 4) experimental protocols. The screening session consisted of a 30‐second all‐out sprint (*Wingate*), a blood lactate profile and an incremental test to exhaustion to determine VO_2max_. Familiarization to the experimental protocol consisted of a 4 hours bout of low‐intensity cycling including three 30‐second maximal sprint efforts toward the end of the first, second, and third hour (*E&S*). On experimental days, the cyclists performed in a randomized manner *E&S* or 4 hours of low‐intensity cycling without sprinting (*E*). Prior to all visits at the laboratory, participants were instructed to refrain from intense exercise, caffeine, beta‐alanine, and bicarbonate for at least 24 hours. Prior to the first experimental session, participants were also instructed to register food intake during the 24 hours leading up to the session, including time of consumption, and were instructed to repeat this prior to the second experimental session 24 hours. Each visit to the laboratory was separated by 4‐9 days and started at the same time of day (~8.00 AM), in a controlled environmental condition (16‐21°C and 20%‐35% relative humidity), with a fan ensuring air circulation.

### 2.3 Experimental protocols

#### 2.3.1 Blood and muscle sampling

Upon arrival, participants rested in the supine position for 30 minutes prior to venous blood sampling from the antecubital vein and muscle biopsy sampling from the m. Vastus Lateralis of a randomized leg, performed under local anesthesia (~2 mL Lidokain, Mylan Dublin, Ireland) using the micro‐biopsy technique (Bard Magnum, Bard Nordic, Helsingør, Denmark) with a 14‐gauge needle (Medax medical devices, Poggio Rusco, Italy). Blood and muscle samples were collected before (Pre), 20‐minute after (Post), and 3 hours after (3h) each experimental protocol. The first muscle biopsy was sampled one third of the distance from the patella to anterior superior iliac spine, with subsequent biopsies being sampled approximately 2 cm proximal to the previous sample. Blood was collected in 9‐mL vacuettes containing Z Serum Clot Activator for serum (Greiner bio‐one GmbH, Austria). After 30 minutes, collected blood was spun at 2600 rpm for 15 minutes on a KUBOTA 2420 (JZ4725‐M000, Tokyo, Japan) and serum was separated from red blood cells, frozen, and stored at − 80°C until further analysis.

#### 2.3.2 Four‐hour exercise protocols with and without sprinting

The *E&S* protocol consisted of 4‐hour cycling at a power output equivalent to 50% of VO_2max_, with 3 × 30‐second maximal sprints interspersed by 4‐minute recovery (1 minutes completely rest and 3‐minute cycling at 100 W) 41 minutes into every hour during the first 3 hours. No sprinting was performed during the last hour (Figure 1). The *E* protocol consisted of 4‐hour cycling at power output equivalent to 50% of power output at VO_2max_. Overall, the average power output was 182 ± 4 W (mean ± SD) and 182 ± 4 W in *E&S* and *E*, respectively. Power output at 50% of VO_2max_ was calculated using submaximal values from the blood lactate profile test together with data from the VO_2max_ test. However, to ensure work‐matched protocols, *E&S* involved slightly higher power output during steady‐state periods (*E&S*: 186 ± 5 W vs *E*: 182 ± 4 W), as caused by the 4‐minute recovery periods between sprints. As reported in a previous paper,^7^ mean power output during each of the three sets of 30‐second sprints in E&S was 787 ± 66 W (1. Set), 782 ± 59 W (2. Set), and 772 ± 60 W (3. Set), equivalent to 93 ± 1%, 92 ± 1%, and 91% ± 1% of values obtained during the all‐out Wingate test. Metabolic cost of cycling increased by 5 ± 1% and 6 ± 1% from the first hour to the last hour of cycling in *E&S* and *E*, respectively, with no difference between protocols, coinciding with oxygen consumption increasing from 50 ± 1% to 53 ± 1% of VO_2max_ in *E&S* and from 48 ± 1% to 51 ± 1% of VO_2max_ in *E*. The two experimental protocols were separated by 6 ± 2 d and were performed in a randomized order.

#### 2.3.3 Isokinetic knee extension for evaluation of recovery

To evaluate recovery of muscle strength after *E&S* and *E*,^23^ one‐legged isokinetic knee extension was performed Pre, Post, 3 hours, and 24 hours after (24h) experimental protocols, using the leg that was not exposed to biopsy sampling. Isokinetic torque was evaluated at 60, 180, and 240°seconds^−1^ using an isokinetic dynamometer (Humac Norm, Computer sports Medicine Inc USA). Subjects performed 3 familiarization trials on both legs prior to experimental protocols. Peak torque of the best repetition was analyzed using the software (HUMAC 2015 v.15, Computer Sports Medicine Inc).

#### 2.3.4 Food and liquid consumption

During familiarization trials, participants consumed water, energy drink, and gels without caffeine (Squeezy Sports Nutrition GmbH, Germany) ad libitum to prevent dehydration and glycogen depletion. Consumption was recorded and duplicated during subsequent experimental sessions. Participants consumed 3.2 ± 0.1 L and 3.2 ± 0.1 L of energy drink and water, during *E&S* and *E*, amounting to 277 ± 17 g and 274 ± 15 g of carbohydrate, respectively. After completion of experimental tests (and after the first post‐exercise muscle biopsy), participants received a body mass standardized meal 30 minutes after exercise, consisting of > 500 mL Chocolate Milk (Sjokomelk, Tine, Norway) and Fruit Müsli (Fruktmüsli, First Price, Germany), amounting to 0.36 g/kg protein, 1.17 g/kg carbohydrate, and 0.16 g/kg fat. Participants otherwise rested at the location and were only allowed to consume water during the preceding 3 hours.

### 2.4 Blood and muscle analyses

Serum concentrations of total testosterone, cortisol, GH, sex hormone‐binding globulin (SHBG), and IGF1 were measured using an Immulite 1000 ExUs edition (Siemens, USA) using kits from the Immulite Immunoassay System Menu (Siemens Medical Solutions Diagnostics, NY, USA). The ratio between free testosterone and SHBG was calculated from this.

Total RNA was extracted from muscle tissue using a combination of phase separation and silica‐column clean‐up. Muscle tissue (**~**30 mg) was homogenized in 200 μL of TRIzol® Reagent (Invitrogen, Life technologies AS, Oslo, Norway), using 0.5 mm RNAse‐free Zirconium Oxide beads and a bead homogenizer (Bullet Blender, Next Advanced, Averill Park, NY, USA), according to manufacturer's instructions, as previously described.^24^ Following homogenization, TRIzol® Reagent was added to a total volume of 1 mL and the homogenate was vortexed and incubated at room temperature for 5 minutes, after which 200 μL of chloroform was added, followed by 3 minutes of incubation and subsequent phase separation by centrifugation (12 000 g, 10 minutes, 4°C). Four hundred microliters of the aqueous phase mixed with an equal volume of 100% ethanol and incubated 10 minutes at room temperature on a silica spin‐column (Zymo‐Spin™ IIC, Zymo Research, Irvin, USA). Following brief centrifugation, the flow‐through was discarded and the column was washed by centrifugation once with RWT buffer and twice with RPE buffer (Qiagen Nordic, Oslo, Norway). RNA was eluted from the column in TE buffer heated to 60°C by centrifugation. RNA purity and quantity were assessed by evaluation of absorbance at 230, 260, and 280 nm using a micro‐volume spectrophotometer (NanoDrop 2000, Thermo Scientific, USA).

Samples were reverse transcribed in duplicates (500 ng total RNA) using SuperScript® IV Reverse Transcriptase (Invitrogen, Life technologies AS, Oslo, Norway), using anchored Oligo‐dT and random hexamer primers (Thermo Scientific, Life technologies AS, Oslo, Norway), according to manufacturer's instructions, as previously described.^24^ Real‐time RT‐PCR was performed on 2 μL cDNA (1:50 dilution) in a 10 μL reaction volume, using 2X SYBR® Select Master Mix (Applied Biosystems, Life technologies AS, Oslo, Norway) and specific primers added at a 0.5 μmol/L final concentration, using a fast‐cycling real‐time detection system using Applied Biosystems™ QuantStudio 5 Real‐Time PCR System (Thermo Fischer Scientific). Cycling consisted of 40 cycles; three seconds at 95°C followed by 30 seconds 60°C. Melt‐curve analysis was performed for all reactions to verify single product amplification. Real‐time RT‐PCR parameters are presented in Table 2.

**TABLE 2:** Sequences of qRT‐PCR primers utilized for analysis of mRNA abundance

Gene	Primers for qRT‐PCR		Efficiency	Cq
	Forward primer	Reverse primer	Mean ± SD	Mean ± SD
β2‐m	TGACTTTGTCACAGCCCAAGA	CGGCATCTTCAAACCTCCATGA	1.98 ± 0.01	23.2 ± 0.3
CLCN1	TTCAGCGCCTTTGTGTTTCG	AATCCCGATGGCAGCAAAAG	1.82 ± 0.01	29.7 ± 1.2
IGF1	ATGTATTGCGCACCCCTCAA	GTACTTCCTTCTGGGTCTTGGG	1.86 ± 0.01	28.9 ± 0.6
MSTN	AGGAGAAGATGGGCTGAATCC	CCCTTCTGGATCTTTTTGGTGTG	1.91 ± 0.01	32.4 ± 0.9
ATP1A1	ATCCTTGAGTACACCTGGCTTG	TTTCCTTGCCATGCGTTTGG	1.62 ± 0.01	32.6 ± 0.8
ATP1B1	ATTTTGGACTGGGCAACTCC	ATTTGGGCTGCAGGAGTTTG	2.09 ± 0.01	23.6 ± 0.4
ATP1A2	TTCCTCGGGGCTTCAAATTC	ATGAGCCCCACAAAGCAAAG	2.16 ± 0.01	23.1 ± 0.6
SLC9A1	TCCATGCAAGTGCTGTTTGG	TTCTTCTGTACAGGCAGCAGAG	1.87 ± 0.01	31.0 ± 0.6
PDK4	CCAGACCAACCAATTCACATCG	TTCAACTGTTGCCCGCATTG	2.06 ± 0.01	25.2 ± 2.0
PGC1αs1	TATGGAGTGACATCGAGTGTGC	ACCCAGAAAGCTGTCTGTATCC	1.94 ± 0.01	26.2 ± 0.6
PGC1αs4	TGTGCCATATCTTCCAGTGACC	TGCAGTTCCAGAGAGTTCCAC	2.06 ± 0.01	27.5 ± 1.3
PPIA	AAGGGTTCCTGCTTTCACAG	TGTGAAGTCACCACCCTGAC	1.66 ± 0.01	25.6 ± 0.4
TBP	AACAGGTGCTAAAGTCAGAGCAG	ACGTCGTCTTCCTGAATCC	1.87 ± 0.01	30.8 ± 0.4
TFAM	AAAGCTCAGAACCCAGATGC	AATCAGGAAGTTCCCTCCAACG	2.06 ± 0.01	27.0 ± 0.3
THBS1	AACAAACAGGTGTGCAAGCC	ACTTGGCGTTCTTGTTGCAG	1.93 ± 0.01	29.5 ± 2.0
VEGFA	CCTGCAAAAACACAGACTCG	CTCGGCTTGTCACATCTGC	1.99 ± 0.01	26.2 ± 0.9

Note

Abbreviations: ATP1A1, Na^+^‐K^+^ α1; ATP1A2, Na^+^‐K^+^ α2; ATP1B1, Na^+^‐K^+^ β1; CLC‐1, chloride voltage‐gated channel 1; IGF1, insulin‐like growth factor 1; PDK4, pyruvate dehydrogenase kinase 4; PGC‐1αs1, peroxisome proliferator‐activated receptor gamma coactivator‐1α splice 1; PGC‐1αs4, peroxisome proliferator‐activated receptor gamma coactivator‐1α splice 4; RPL32, ribosomal protein L32; SLC9A1/NHE1, sodium‐hydrogen exchanger 1; TBP, TATA‐box binding protein; TFAM, mitochondrial transcription factor A; THSB1, thrombospondin; VEGFA, vascular endothelial growth factor A; β2‐m, β2 microglobulin.

Average primer efficiencies and Cq‐values for all reactions are given. Values are mean ± SD.

Quantification cycles (Cq) were determined using the second derivate method from raw fluorescence data, exported from the Applied Biosystems software and analyzed using the qpcR‐library.^25^ Primer efficiency values were estimated from single reactions and averaged for each primer pair.^25^ Gene abundance data were efficiency corrected, and analysis was done using log‐transformed values. In order to control for RNA quantity in cDNA synthesis and subsequent dilution, suitable reference genes were determined from systematic evaluation of 12 transcripts (sequences available on request) with modification for the present repeated‐measures study design, as previously described.^24^ Beta‐2‐microbulin (B2m), TATA‐box‐binding protein (TBP) and peptidyl‐prolyl cis‐trans isomerase A (PPIA), as their abundance did not change with sampling time‐points or exercise condition. Normalization of target genes was thus performed using the geometric average of these three internal reference genes as described previously.^24^

### 2.5 Statistics

For Log2 fold changes in mRNA abundance and blood hormone levels, a marginal model was applied, wherein effects of time (Pre, Post, 3h) and exercise condition (*E&S*, *E*) and their interaction were assessed using SPSS‐software version 23. Time and condition were specified as fixed effects. Repeated measures were specified by subject. To compare changes between conditions, a marginal model with baseline values as a co‐variate was used. A significant main effect or interaction was further evaluated by a multiple‐comparison approach with Sidak adjustment. A significance level of 0.05 was applied, and data were expressed as mean ± 95% confidence interval (CI). Gene abundance data are presented as Log2 fold change from pre‐exercise ± 95% CI.

## 3 RESULTS

### 3.1 Gene abundance in m. Vastus lateralis

**FIGURE 2:**
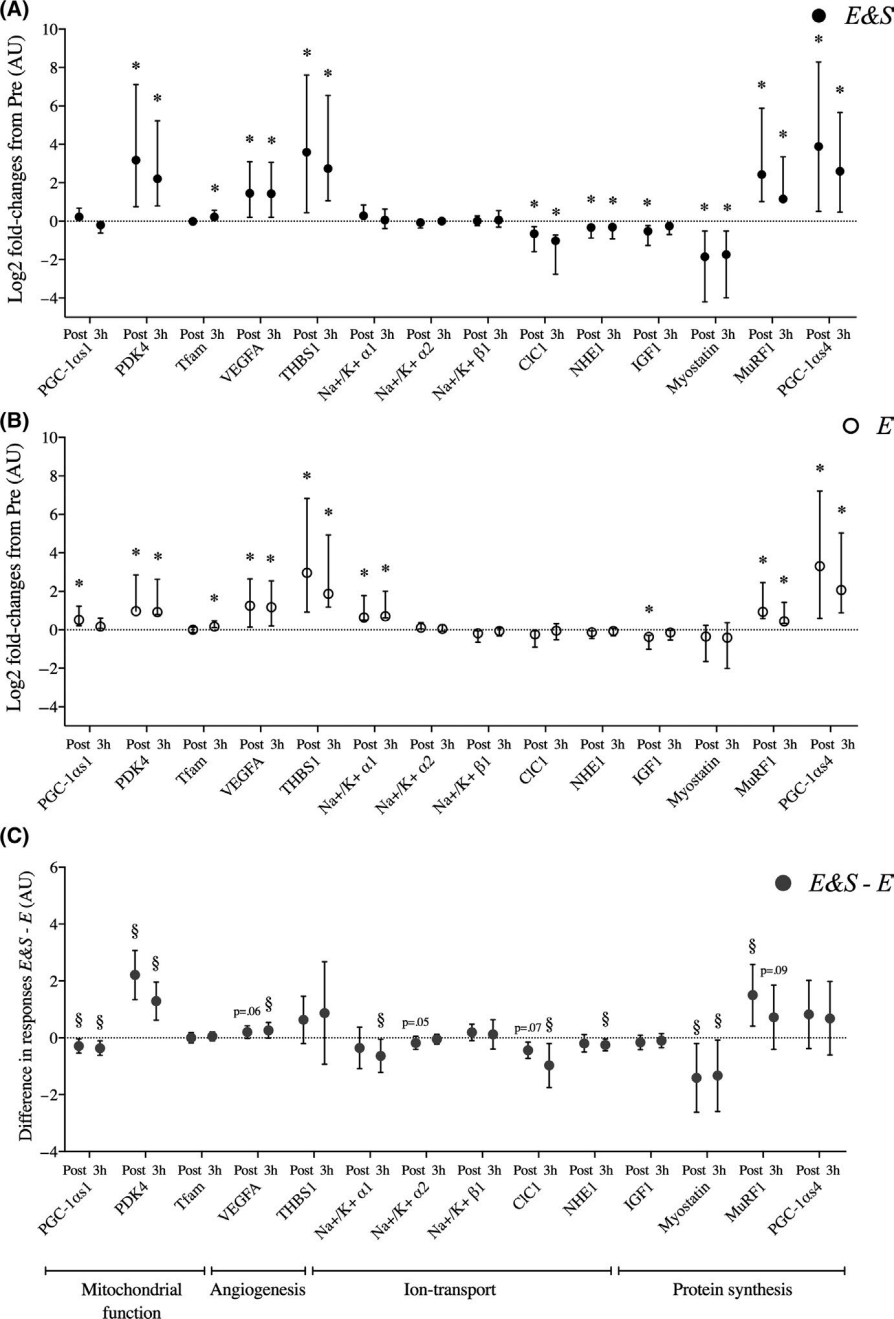
A and B) Effects of 4 h low‐intensity cycling with (E&S, panel A) or without sprint intervals (E, panel B) on mRNA abundances of markers of mitochondrial function, angiogenesis, ion transport, and protein turnover in m. vastus lateralis of elite cyclists, measured directly after (Post) and 3 h exercise (3 h). Values are log2‐fold changes with 95% CI. C, Differences in responses between E&S and E. Markers of mitochondrial function: peroxisome proliferator‐activated receptor gamma coactivator‐1α splice 1 (PGC‐1αs1), pyruvate dehydrogenase kinase 4 (PDK4), mitochondrial transcription factor A (TFAM), angiogenesis: vascular endothelial growth factor A (VEGFA) and thrombospondin (THBS1). Markers of ion transport: Na^+^‐K^+^ α1 (ATP1A1), Na^+^‐K^+^ α2 (ATP1A2), and Na^+^‐K^+^ β1 (ATP1B1), chloride voltage‐gated channel 1 (CLC‐1), sodium‐hydrogen exchanger 1 (SLC9A1/NHE1). Markers of protein synthesis regulation: insulin‐like growth factor 1 (IGF1), myostatin, muscle ring finger 1 (MuRF1), peroxisome proliferator‐activated receptor gamma coactivator‐1α splice 4 (PGC‐1αs4). * indicates significant (*P* < .05) difference from pre‐exercise, § indicates significant (*P* < .05) difference in response between conditions, tendencies to difference in responses are indicated by *P*‐values. n = 12

*E&S* led to different changes in abundance of mRNA on markers related to mitochondrial function, angiogenesis, ion transport, and protein synthesis compared with *E*. For markers of mitochondrial function, *E&S* was not associated with changes in PGC‐1αs1 mRNA (Figure 2A), while *E* led to increased abundances at Post (*P* < .05; Figure 2B), but not at 3h. In effect, *E&S* was associated with reduced PGC‐1αs1 mRNA levels compared with *E* (Figure 2C), measured as changes from Pre to Post (*E&S:* 1.2 ± 0.2 fold vs*E:* 1.5 ± 0.3 fold) and from Pre to 3h (*E&S:* 0.9 ± 0.2 fold vs*E:* 1.2 ± 0.3 fold). For PDK4, *E&S* and *E* led to increased mRNA abundances at both Post and 3h (Figure 2A+B), with responses being more pronounced in *E&S* at both Post (*E&S:* 11.9 ± 8.7‐fold vs*E:* 2.8 ± 2.6‐fold, *P* < .05) and 3h (*E&S:* 6.1 ± 3.9‐fold vs *E:* 2.6 ± 2.4‐fold, *P* < .05; Figure 2C). For both *E&S* and *E*, TFAM was only elevated 3h (both *P* < .05; Figure 2A+B) without differences between conditions (Figure 2C).

For markers of angiogenesis, *E&S* and *E* led to increased abundances of VEGFA and THBS1 mRNA at both Post and 3h (all *P* < .05; Figure 2A+B), with VEGFA responses being more pronounced in *E&S* at 3h (*E&S:* 2.8 ± 0.6‐fold *vs E:* 2.3 ± 0.5‐fold, *P* < .05; Figure 2C).

For markers of ion transportation, *E&S* was associated with suppression of mRNA levels for several components involved in Na^+^, K^+^, and H^+^ transport compared with *E* (Figure 2A+B). While *E&S* had no effect on Na^+^‐K^+^ α1 mRNA levels, *E* led to increased levels at both Post and 3h, resulting in differential responses between the two exercise modalities at 3h (*E&S:* 1.2 ± 0.4‐fold vs*E:* 2.1 ± 1.2‐fold *P* < .05; Figure 2C). *E&S* led to decreased levels of CLC1 mRNA at Post and 3h, with *E* having no effect (Figure 2A+B), resulting in negative changes in *E&S* compared with *E* at 3h (*E&S:* 0.6 ± 0.4‐fold *vs E:* 1.1 ± 0.6‐fold *P* < .05; Figure 2C). Neither of the two exercise modalities affected Na^+^‐K^+^ α2 or Na^+^‐K^+^ β1 mRNA abundances, and there were no differences between conditions.

For markers of protein synthesis, *E&S* and *E* led to reduced IGF1 mRNA abundance at Post (*P* < .05), and increased MuRF1 and PGC‐1αs4 abundances at Post and 3h (*P* < .05; Figure 2A+B). *E&S* only led to decreased myostatin mRNA abundances (*P* < .05; Figure 2A). Overall, *E&S* was associated with reduced myostatin mRNA abundances compared with *E* at Post (*E&S:* 0.3 ± 0.2‐fold vs*E:* 1.3 ± 0.8‐fold, *P* < .05) and 3h (*E&S:* 0.3 ± 0.2‐fold vs*E:* 2.1 ± 2.6‐fold, *P* < .05; Figure 2C), and increased MuRF1 mRNA abundances at Post (*E&S:* 4.5 ± 1.2‐fold vs*E:* 2.3 ± 0.9‐fold, *P* < .05; Figure 2C), measured as changes from Pre.

### 3.2 Blood hormone responses

**FIGURE 3:**
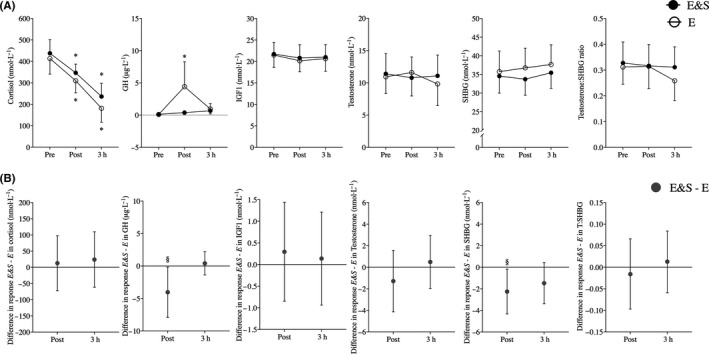
Blood hormone responses to 4‐h low‐intensity exercise with (E&S) or without sprints (E). A, Hormone concentrations in blood measured before (Pre), 20 min after (Post) and 3 h after exercise (3h). B, Differences in absolute changes in blood hormone concentrations between Pre and Post, and Pre and 3h (values are E&S – E). Cortisol, growth hormone (GH), insulin‐like growth factor 1 (IGF1), testosterone, sex hormone‐binding globulin (SHBG), and testosterone:SHBG ratio. * indicates significant (*P* < .05) difference from pre‐exercise, § indicates significant (*P* < .05) difference in response between conditions, mean ± 95% CI, n = 12

Overall, *E&S* and *E* had little impact on hormonal concentrations in blood, with only cortisol showing clear‐cut time‐dependent changes (Figure 3A). For GH, *E&S* led to blunted responses at Post, contrasting the 4.3 ± 4.3 μg/L increase observed in *E*, resulting in negative changes in *E&S* compared with *E* (*P* < .05; Figure 3B). For SHBG, no effect was seen of either condition on blood concentrations (Figure 3A), though *E&S* was associated with negative changes at Post compared with *E* (*P* < .05; Figure 3B), without affecting testosterone:SHBG ratios. For cortisol, IGF1, and testosterone, *E&S* and *E* resulted in similar blood concentration profiles (Figure 3A).

### 3.3 Isokinetic knee extension

**FIGURE 4:**
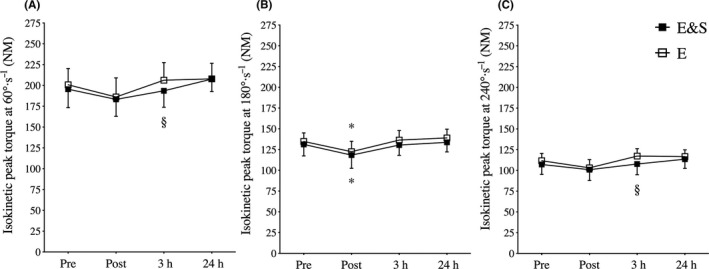
Isokinetic knee extension torque before (Pre), after (Post), 3 h after (3h), and 24 h after exercise (24h). Knee extension was performed at three different speeds: Panel A; 60°·seconds^−1^, Panel B; 180°· seconds^−1^, and Panel C; 240°·seconds^−1^. Mean ± 95% CI, * indicates significant (*P* < .05) difference from pre‐exercise, § indicates significant (*P* < .05) difference from E, n = 12

Immediately after *E&S* and *E* (Post), participants displayed similar reductions in isokinetic knee extension torque at 180°·seconds^−1^ compared with Pre (*P* < .05; Figure 4B), with no changes being evident at 60°·seconds^−1^ or 240°· seconds^−1^. Torque at 180°·seconds^−1^ was recovered 3h exercise in both *E&S* and *E* but torque was lower at 60°· and 240°·seconds^−1^ in *E&S* compared with *E* (*P* < .05; Figure 3 panel C). For both conditions, torque was equally recovered at 24h at all velocities.

## 4 DISCUSSION

The present study investigated the effects of including repeated 30‐seconds sprints during a 4‐hour LIT session on acute mRNA responses in muscle, hormonal responses in blood, and recovery of isokinetic knee extension torque in elite cyclists. In muscle, *E&S* led to augmented increases in mRNA levels of markers of fat metabolism (PDK4), angiogenesis (VEGFA), and muscle hypertrophy (myostatin and MuRF1) compared with *E,* while it was associated with decreased levels of markers of ion transport (Na^+^‐K^+^ α1, CLC1, and NHE1) and blunted responses for a marker of mitochondrial biogenesis (PGC‐1α) compared with *E*. The overall impression is thus that *E&S* led to more pronounced changes in muscle cells, supporting our initial hypotheses. In blood, GH and SHBG showed slightly reduced responses to *E&S* immediately after the exercise compared with *E*, while other endocrine variables changed in similar manners in response to both training modalities at Post and 3h. Muscle strength, measured as isokinetic knee extension torque, was completely restored 24 hours after both *E&S* and *E*.

### 4.1 mRNA abundances in muscle

#### 4.1.1 Mitochondrial function and biogenesis

In the present study, *E&S* led to greater acute increases in PDK4 mRNA in muscle immediately after and 3 hours after the exercise compared with *E*. This was in line with our hypothesis and is supported by previous findings in trained subjects performing a relatively short (~1.5 hours) *E&S* protocol,^10^ and may act to shunt metabolism toward fatty acid metabolism.^26^ PDK4 activity impairs oxidation of carbohydrates through negative regulation of the pyruvate dehydrogenase complex, probably potentiated by excessive glucose utilization during exhaustive exercises such as sprinting.^26^ Unfortunately, we cannot confirm the latter perspective in the present study, as we did not measure muscle glycogen content. However, combined sprint and endurance exercise has previously shown to induce greater depletion of glycogen stores compared with endurance exercise only,^8^ even when the *E&S* protocol is performed over a short time span (1 hour) without carbohydrate feeding.^8^ An interrelationship between PDK4 expression and glucose availability therefore seems likely. Furthermore, sprint exercise acutely increases several genes involved in fat metabolism, including PDK4,^27^ and 6 weeks of sprint training leads to increased activity of enzymes involved in fat metabolism‐trained subjects.^28^ It thus seems plausible to suggest that *E&S* increases the capacity of muscle tissue to metabolize fatty acids, despite continuous carbohydrate feeding during the exercise. However, sprint training has also been shown to inhibit mitochondrial respiration,^29^ but also increase oxygen affinity in mitochondria.^30^ The long‐term oxidative adaptations to sprint training in elite athletes therefore need further investigation.

Neither *E&S* nor *E* led to robust increases in PGC‐1α mRNA in muscle (1.5‐fold), with *E* resulting in slightly larger responses both immediately after and 3 hours after exercise. This contradicted our initial expectations, and also contradicts previous studies, wherein *E&S* has led to rather large increases in PGC‐1α expression (~6‐ to 10‐fold), and larger than LIT exercise without sprints,^8^, ^10^ though this is not found in all previous studies.^9^ Overall, PGC‐1α activity plays a center‐stage role in endurance‐mediated muscular adaptations, such as improved mitochondrial functions, mitochondrial biogenesis, and angiogenesis.^31^ PGC‐1α induces positive regulation of PDK4 and VEGFA mRNA abundance,^31^ which makes the blunted PGC‐1α mRNA responses in *E&S* compared to *E* difficult to explain, as both PDK4 and VEGFA increased in response to exercise, and more so in response to *E&S* immediately after and 3 hours after exercise. This seeming contradiction is further highlighted by the positive effects of combined sprint and LIT exercises on mitochondrial adaptations over time.^9^ The design of our study offers a few potential explanations to these apparent contradictions. First, the participants held a higher fitness level than those of previous studies^8^, ^9^, ^10^; that is, they were elite cyclists and not merely well‐trained. This may have affected muscle cellular responses to exercise, a phenomenon that is even seen in previously untrained individuals after conduction of as few as < 14 endurance exercises, reducing acute PGC‐1α mRNA responses from 7‐fold to ~ 2‐fold increases.^32^ Second, the blunted PGC‐1α mRNA responses may be an artifact of the timing of muscle biopsy sampling, which was performed ~ 3.5 hours (Post) and ~ 7.5 hours (3 h) after finalization of the first sprint‐set in the *E&S* group. Following such relatively prolonged time lags, any initial increases in PGC1‐α expression may have been corrected through feedback mechanisms. Adding to this, performing several consecutive sets of sprinting within a limited timeframe may itself be associated with blunted responses at the mRNA level. Such blunting has previously been observed for PGC‐1α mRNA, albeit over a much longer period (1‐14 sessions).^32^ At present, we do not know how PGC1‐α mRNA response dynamics are affected by repeated sets of sprint intervals during LIT and can thus not exclude that PGC‐1α mRNA responses peaked during the four hours of exercise. Importantly, in Gunnarsson et al (2019), PGC‐1α mRNA responses did not differ between LIT exercises (1 hour) performed with and without inclusion of sprints and eight weeks of LIT and sprints still led to increased mitochondrial protein content.^9^ This supports the notion that acute PGC‐1α mRNA abundance does not necessarily coincide with longitudinal changes in PGC‐1α protein activity.^33^ Third, PGC‐1α activity is interconnected with carbohydrate availability and increases in response to decreased muscle glycogen levels.^34^ As combined sprint and LIT protocols seem to induce greater glycogen depletion than LIT,^8^ this may have contributed to the previously observed increases in PGC1‐α mRNA abundances.^8^, ^10^ In the present study, we sought to increase the ecological validity of our protocol by allowing participants to ingest exogenous glucose during exercise, thus likely avoiding pronounced glycogen depletion. This may have contributed to the relatively small changes in PGC‐1α mRNA abundance (1.5‐fold). The relatively miniscule changes in PGC1‐α mRNA abundances observed in response to both *E&S* and *E* in the current study warrant further investigation into the mitochondrial responses to exercise in elite athletes.

#### 4.1.2 Angiogenesis

*E&S* and *E* led to marked angiogenic responses in muscle, evident as increased abundances of VEGFA and THBS1 mRNA, resembling typical observations made after endurance exercise, leading to capillary growth after training.^35^ For VEGFA, the response after *E&S* was more pronounced 3 hours after exercise, suggesting that adding sprint intervals to prolonged LIT may exert greater effects on angiogenic processes than low‐intensity exercise alone, possibly leading to greater vascularization over time. Notably, the augmented VEGFA response seen after *E&S* in the present study contradicts data from recent studies,^8^, ^10^ wherein no beneficial effect was seen of including sprint into a LIT session on VEGFA mRNA abundances in muscle of trained subjects. In both studies, the duration of the exercise was much shorter than in the present study (1.5 hours vs 4 hours),^8^, ^10^ suggesting that the potentially positive effects of adding sprints to LIT cycling on angiogenesis may depend on the overall duration of the exercise.

#### 4.1.3 Ion transport

*E&S* and *E* led to differential changes in mRNA abundances of Na^+^‐K^+^ α1, NHE1, and CLC‐1, with *E&S* leading to reduced levels of all transcripts 3 hours after exercise, measured as changes from Pre to Post and/or 3h. While it could be interpreted as a general reduction in ion transport capacity after *E&S*, this seems to be an invalid biological interpretation. As previously reviewed,^13^ most studies find increased ion transport capacity in muscle after sprint training, both with and without changes in protein content, which may play a critical role for postponing fatigue. Therefore, we argue that the reduced levels in *E&S* may be due to feedback mechanisms, wherein expression of genes involved in ion fluxes show temporal reduction, perhaps relating to allocation of cellular resources toward first‐line adaptations such as cellular repair. While this remains speculative, genes such as NHE1 are known to show delayed exercise‐induced increases in mRNA abundances (24‐48 hours) in recreationally active men, with only small increases being detected during the initial 9 hours.^36^ While ion transporters such as Na^+^‐K^+^ ATPases and NHE1 reset homeostasis after muscular contractions, CLC‐1 regulates muscle excitability during contractions to failure, predominantly in type II muscle fibers.^37^ In humans, CLC‐1 content is higher in type II fibers, with protein abundances correlating negatively with exercise performance.^38^ In the present study, the negative effects of *E&S* on CLC‐1 mRNA abundances may thus relate to excessive activation of type II muscle fibers during sprints, which over time may lead to altered CLC‐1 protein content. In contrast to this hypothesis, Thomassen et al (2018)^38^ did not reveal any such changes in CLC‐1 content in response to 4 weeks of *E&S*. It is thus reasonable to question the biological significance of our observation, which may instead result from a negative feedback response, as previously discussed. However, compared to Thomassen et al (2018),^38^ our *E&S* study protocol was more physiologically demanding (<1 hours vs 4 hours). Given the plausible role of CLC‐1 in regulating muscle excitability,^37^ it remains an intriguing possibility that *E&S*, and perhaps type II fiber‐activating endurance training in general, alters CLC‐1 biology over time, acting to improve fatigue‐resistance and possibly high‐intensity endurance performance in elite cyclists.

#### 4.1.4 Muscle protein turnover

*E&S* and *E* led to differential responses for myostatin and MuRF1 mRNA, with *E&S* being associated with decreased myostatin abundances immediately after and 3 hours after exercise, and more pronounced increases in MuRF1 immediately after exercise. This suggests increased protein turnover in *E&S* compared with *E*, as reduced myostatin levels typically promote muscle protein synthesis^14^ and increased MuRF1 levels increase rates of proteolysis.^39^, ^40^ This corroborates with acute changes seen after both resistance and endurance exercise.^40^ Resistance exercise is typically associated with increased transcription of genes involved in protein synthesis, leading to hypertrophy,^41^ while endurance exercise is associated with proteolytic gene responses,^40^ potentially explaining the reduced muscle fiber size seen in endurance‐trained athletes.^42^ In the present study, the augmented myostatin and MuRF1 responses to *E&S* (compared to *E*) may be due to the substantially greater activation of type II fibers during sprinting.^12^ Further research is warranted to investigate the effects of repeated sessions of *E&S* on changes in muscle mass and phenotype.

### 4.2 Blood hormone response

*E&S* and *E* led to similar changes in blood concentrations of cortisol, IGF1, and testosterone, while *E&S* was associated with blunted responses compared to *E* for GH and SHBG immediately after the exercise but not at 3 hours. The absence of testosterone responses in the present study was in contrast to our initial hypothesis, as it contrasts previous findings in trained athletes, evident as acute increases in testosterone after both LIT exercise (2 hours at ~ 55% VO_2max_) and repeated 30‐second sprinting.^17^ However, the high fitness level of our participants may have affected blood hormonal responses, as higher fitness increases the threshold intensity at which these responses occur.^43^ Indeed, elite cyclists seem to display only small degrees of metabolic stress after habitual cycling for 4 hours, as compared to exhaustive endurance exercise.^44^

We previously showed that the present *E&S* protocol leads to elevated lactate levels during the course of the exercise compared to *E*,^7^ suggesting that it is associated with greater metabolic strain.^45^ GH and cortisol levels in blood are also regarded as biomarkers of such metabolic strain,^21^, ^46^ and hence tend to increase in an intensity‐dependent manner.^17^ It was thus surprising that GH concentrations in blood did not increase after *E&S*, while it increased after *E* (~5 ug/L), with the latter response resembling observations after 2 hours of low‐intensity exercise (~4 ug/L).^17^ The blunted GH response in *E&S* compared with *E,* contrasts previously reported effects of repeated sprinting, which typically leads to elevated GH levels.^15^, ^27^ Arguably, this aberrancy may be due to the timing of blood sampling, as blood was collected ~ 3.5 hours after the first sprint and ~ 90 minutes after the last sprint. Sprint‐induced increases in GH levels typically return to baseline within 60‐80 minutes.^17^, ^27^ Hence, temporal fluctuations in markers of metabolic strain in response to sprinting could have occurred during *E&S*. As for cortisol, blood levels decreased steadily throughout the intervention after both *E&S* and *E*. While this also contrasts previously reported increases after sprint exercise^17^ and may also have been affected by the timing of blood, other factors were likely more determining for the overall negative response. Cortisol levels vary with food intake and also varies in a circadian manner, whereby it increases in periods of fasting, that is throughout the night, and decreases throughout the day.^47^ Hence, the steadily decreasing cortisol levels were likely associated with the continuous intake of carbohydrates during the intervention, along with its prolonged duration, starting at 8 AM and lasting for 7 hours (corresponding to blood sampling at 3 hours). Overall, the low levels of GH and the decreasing levels of cortisol measured after both conditions suggest that the present *E&S* and *E* protocols were associated with relatively low degrees of metabolic strain in elite cyclists, with no evidence for a prolonged stress response in the three hours following exercise.

The decreased SHBG levels observed immediately after *E&S* compared with *E* may have affected testosterone biology. Albeit speculatively, this may have led to increased levels of free testosterone in blood, as less SHBG would be present to bind testosterone, resulting in augmented testosterone‐induced anabolic signaling.^16^ Inclusion of sprint intervals during low‐intensity exercise may thus counteract the impaired testosterone signaling typically seen in athletes performing large volumes of endurance training, which is associated with reduced testosterone levels.^19^ This may have beneficial effects on physiological responses to training, facilitating muscle plasticity,^16^ and erythropoiesis.^20^ Unfortunately, we did not measure free testosterone in the present study, nor did we find clues to support reduced free testosterone levels in blood, with testosterone:SHBG ratios remaining unchanged after both training protocols.

### 4.3 Recovery of isokinetic torque

*E&S* and *E* led to similar reductions in isokinetic knee extension torque measured immediately after the exercise (Post), and both sessions were associated with complete recovery the following day (24 hours). In trained athletes, reductions in peak torque are typically observed after periods of relatively large loads of aerobic endurance exercise with inadequate periods of recovery.^23^ Notably, at 3 hours, *E&S* was associated with reduced torque both at the lowest and highest contraction velocities compared with *E*, suggesting greater muscular fatigue in both slow and fast motor units during the initial phase of the recovery period.^23^ However, as this reduction was temporary and not evident after 24 hours, we suggest that 30‐second sprint intervals included in LIT do not lead to excessive need for recovery in elite athletes, a perspective that is supported by the lack of marked endocrine stress responses. To the contrary, it seems to be a feasible and readily available training strategy that does not reduce muscle performance on consecutive days, though this needs to be confirmed by studies investigating the effects of repeated *E&S* session over prolonged time‐periods.

## 5 CONCLUSION

Inclusion of 30‐seconds sprints during a prolonged LIT session induced greater mRNA responses for markers of fat metabolism (PDK4), angiogenesis (VEGFA), and protein turnover (MuRF1 and Myostatin), but decreased mRNA levels for markers of ion transport (Na^+^‐K^+^ α1, CLC1, and NHE1) and mitochondrial biogenesis (PGC‐1α) in m. vastus lateralis of elite cyclists compared with work‐matched LIT cycling. There were small differences between *E&S* and *E* in endocrine stress responses, but muscle strength in thigh muscles was fully recovered 24 hours after both conditions. Inclusion of sprints to prolonged LIT exercises might thus be an efficient training strategy for elite athletes, potentially leading to beneficial adaptations in skeletal muscle without affecting muscle performance on consecutive days.

## 6 PERSPECTIVE

On a general basis, the present study adds valuable insight into the acute effects of LIT with and without inclusion of sprint intervals in elite endurance athletes. At present, such knowledge is scarce, likely due to difficulties with recruiting top‐level athletes to research studies, especially upon request for muscle biopsies. Utilization of the minimally invasive micro‐biopsy procedure should open the avenue for more such studies in the future, minimizing risks for adverse events while simultaneously providing valid and reliable data. For elite athletes, inclusion of sprint intervals to prolonged LIT session seems to offer an effective exercise strategy for simultaneous development of endurance performance and sprint ability in the same exercise session, potentially increasing the performance‐enhancing effects of training periods focusing on LIT. Future studies should elaborate on the longitudinal effects of such training, including performance as well as aspects of muscle biology such as mitochondrial biogenesis and function, and possible changes in muscle mass and phenotype.

## CONFLICT OF INTEREST

None.
